# Practical Sociology: Ill-Health as a Cause of Pauperism

**Published:** 1908-02-29

**Authors:** A. D. Edwards

**Affiliations:** Resident Medical Officer, Cardiff Union Workhouse.


### HOSPITAL ADMINISTRATION.

### CONSTRUCTION AND ECONOMICS.

### PRACTICAL SOCIOLOGY: ILL-HEALTH AS A CAUSE OF PAUPERISM.

By A. D. EDWARDS, M.B., B.S., B.Sc., D.P.H., Resident Medical Officer, Cardiff Union Workhouse.

The problem of pauperism is one that is receiving ever-increasing attention from present-day economists, and whilst legislation in the past has been directed mainly to the alleviation of distress, there is to-day a desire on the part of our legislators to deal with the problem on a more rational basis—that of prevention.

It is a recognised fact that one of the chief causes of pauperism is ill-health; and with the steadily increasing efficiency of our public health methods, it is probable that the amount of pauperism due to this cause will be decreased to an appreciable degree in the future. State medicine is still in its infancy. Not in this generation, nor perhaps in the next, will it attain to its full power; but gradually it will raise the standard of the national health, decrease the case-incidence and mortality of some diseases, and eliminate others altogether. Any discussion of pauperism by our legislators would be incomplete did it not take into account the effect that State medicine will have on it by decreasing one of the chief causes.

The following table has been made from a number of inmates at the Cardiff Union Workhouse and Infirmary, the pauperising diseases being briefly dealt with, and the possibility of their prevention in future generations. As the failure of the wage-earner to continue at work must be regarded as the determining cause of pauperism, the statistics have been taken from a number of adult males, and only those men are included who can be classed as permanent paupers. By the term "permanent pauper" is meant one who is likely to require State maintenance during the rest of his life.

The proportion of pauperising diseases in any Union will be influenced largely by the local conditions obtaining in that Union; thus in a district in which the case-incidence of phthisis is great, that disease will figure more largely in the returns; and similarly, in a great manufacturing centre, the relative number of men who have become paupers on account of occupation-diseases will be greater than in an agricultural district. In the present case—that of Cardiff—the fact that the Union includes a great seaport has an appreciable influence on the proportion referred to. Probably, slight physical unfitness is more apt to shorten the wage-earning years of a sea-faring man than those of any other large class of workers. This is due in some measure to the great importance of good eyesight in the work of a sailor; and thus in the Cardiff Union the number of men incapacitated by eye affections is relatively large.

Another factor which is beginning to exert a marked influence on the number of seamen paupers in the Cardiff Union Workhouse is the new Compensation Act. Seamen suffering from the less serious physical infirmities, who formerly could obtain employment at sea, are now rejected by the examining medical man, and as a result seek State maintenance at a considerably earlier age than they formerly did.

The reference thus made to a few of the factors in the Cardiff Union will indicate that the varying conditions in each Union must be taken into consideration in dealing with the total pauperism of the State, but the remarks following the tables given for the Cardiff Union Workhouse and Hospital may be taken as applicable, with a few modifications, to the average workhouse and workhouse infirmary.

Table A.

| | |
|---|---|
| No. of adult males in Workhouse and Infirmary ... | 335 |
| No. of adult males who may be classed as "Permanent" Paupers ... ... ... ... ... | 237 |
| No. of adult males who may be classed as "Temporary" Paupers* ... ... ... ... ... | 98 |

Table B.

*The Causes of Permanent Pauperism of 237 adult males.*

| | |
|---|---|
| Phthisis and other forms of "Consumption" | 21 |
| † Heart disease ... ... ... ... ... ... | 23 |
| Chronic bronchitis ... ... ... ... | 16 |
| Ulcerations and allied conditions ... ... | 11 |
| Rheumatism, sciatica, etc. ... ... ... | 21 |
| Senility ... ... ... ... ... ... ... | 29 |
| Paralyses ... ... ... ... ... ... ... | 8 |
| Other nerve conditions ... ... ... ... | 3 |
| Uro-genital disease ... ... ... ... ... | 11 |
| Eye troubles ... ... ... ... ... ... | 37 |
| Permanent injuries ... ... ... ... ... | 32 |
| (Joints 13, limbs 14, head injuries 5.) | |
| Inoperable hernia ... ... ... ... ... | 5 |
| Malignant disease (cancer, etc.) ... ... | 4 |
| Deafness ... ... ... ... ... ... ... | 5 |
| Unclassified ... ... ... ... ... ... | 13 |
| Total ... ... ... ... ... | 237 |

In the case of phthisis we may be most sanguine in expecting a marked decrease. Forty years ago the proportion of phthisis cases to the population was twice as great as it is at present, and the remarkable decrease during the past few decades has resulted from but a very imperfect application of the principles of hygiene. It is certain that the earlier diagnosis of cases, their notification and isolation, will result not only in a still more remarkable diminution, but in the abolition of disease. The early isolation of cases is at present a difficult matter. Patients in the first stages of consumption are notably sanguine, and this, together with the fact that workhouse infirmaries are still undeservedly unpopular among the poor, results in the phthisis patient remaining at home, a focus of infection for the other occupants of the house. A wise provision has been made whereby increased money may be granted as out-door relief in cases where a consumptive patient occupies a separate room, but, chiefly on account of lack of accommodation in the houses of the poor, this provision is seldom made use of. And because the patient is not removed and isolated in the early stages of the disease, the phthisis wards of the infirmaries are filled

* Temporarily unable to support themselves on account of ill-health or inability to find work. † See Text.

with consumptives in whom the disease is too far advanced for any great hope of cure; all that can be done for the majority of such cases is to give them medical and nursing attention such as may relieve their sufferings. Hitherto the State had used but little the means at its command to check the spread of disease, and there are still some Union infirmaries in which phthisis is treated in general wards. How many ordinary patients in such wards have developed phthisis ultimately is an unpleasant matter to consider. But these things are being remedied. There are sure signs that the State and the local authorities are becoming conscious of their responsibilities in the matter of infectious diseases, and in no part of Public Health administration is the outlook more favourable than in that dealing with phthisis.

Fifty-nine per cent. of the cardiac cases had mitral lesions, with rheumatic fever, usually in childhood, as the determining cause. The more careful treatment of rheumatic fever, and the greater insistence of the modern school of medical men on prolonged and absolute rest in the treatment of the heart complications, are already decreasing the proportion of heart-disease cases arising from this cause, so that here also we may look for a diminution in the number of persons who become paupers on account of the disease. The remaining 41 per cent. were chiefly cases of aortic disease, over 50 per cent. of whom gave a history or showed signs of past syphilis. The question of venereal disease will be mentioned later.

Of the 16 cases of chronic bronchitis, nearly all were patients in whom repeated attacks had resulted in a chronic condition with accompanying heart strain. The prospect of decreasing the case-incidence of bronchitis is not so good, but we may well hope that as people become educated to a true appreciation of the value of fresh air, and as the facilities for obtaining it improve, there will be a decided diminution in the number of cases.

The usual cause of ulceration and similar conditions becoming permanent is neglect on the part of the patient. The average working man regards an ulcer as a very slight trouble, and allows it to continue for months or years before he acts on medical advice and undergoes thorough treatment. Thorough treatment in the early stages would result in a complete cure in the very great majority of cases, and would thus prevent the condition becoming chronic and permanent.

To hope for a reduction in the case-incidence of chronic rheumatoid and arthritic complaints one must have great faith in the progress of modern medicine; but in view of the possibility of the conditions being microbic, the hope is not altogether unjustified.

For the prevention of senility as a cause of pauperism we cannot look to medical science; the question will, perhaps, be dealt with in the near future by the institution of old-age pensions. The number of such cases in the present table—29—appears small, but many of the paupers otherwise classed are, at their present ages, senile as well. Disease, however, caused their pauperism at a time when, free from the determining ailment, they would have been self-supporting.

A large number of the paralytic patients, as also of the next class (urogenital), are due to former insufficiently treated venereal trouble. How far this will be improved in future generations it is impossible to forecast. At present there is practically no attempt at checking the spread of venereal disease, the only influence towards that end being the presumably increasing chastity of the race. There is no doubt that these diseases are spread to some extent by the tramp class, who are unable to obtain the necessary prolonged treatment, for the policy of all Boards of Guardians is to prevent the admission into their infirmaries of casuals suffering from the complaint.

In the other classes of disease there is practically no prospect of diminution. The mental cases (11) retained in the institution make a very small percentage of such cases admitted, for nearly all are sent to asylums. Of the eye conditions 16 per cent. are due to old injuries, most of the remainder being cataracts and failing accommodation.

Having indicated briefly the degree to which these diseases, as such, may be prevented in the future, it would be well to consider the influence of two factors as causes, or predisposing causes, of disease—*i.e.* alcohol and bad feeding.

So well recognised is the ruinous effect on the system of continued over-indulgence in alcohol, that it is unnecessary to do more than mention its threefold attack on the national health. Firstly, it acts as a direct cause of disease, as in cirrhosis of the liver; secondly, as a predisposing cause by lessening the natural resistance of the body to disease, and thus preparing the soil for the seeds of consumption and other conditions; and thirdly, it has a dire effect on the progeny of its victims, the children of whom are notably deficient in resisting power to disease.

The old general factor to be noted is bad feeding. These two factors are probably interdependent to a great degree. Alcoholism, apart from its effect in increasing disease-caused pauperism, has a direct economic effect in causing non-disease pauperism. But before the drunkard becomes a pauper there is often a period during which his family suffers from insufficient food, and this semi-starvation of children is one of the strongest possible predisposing causes of future ill-health.

The question may be considered, briefly, whether these two great general factors of disease will be rendered less powerful in future generations. It is probable that the amelioration of the conditions of life among the "working classes," the improvement in the general standard of their living, the sweeping away of their slums, and the general brightening of their homes will result in a great decrease in habitual drunkenness; and this amelioration, by improving the standard of feeding, together with the growing usefulness of State medicine in the care of the child health of the nation, will surely bring about a great improvement in the physical stamina of the generations to come, with a resultant decrease of disease-caused pauperism.

These hopes may appear to be Utopian, but we, watching the progress of preventive medicine, may justifiably cherish them. Such progress may be necessarily slow; nevertheless, it will be sure.

